# Heterogeneous effect of saxagliptin on glucose fluctuation and β-cell function in T1DM: a multicentre, randomised trial

**DOI:** 10.1038/s41387-026-00411-3

**Published:** 2026-03-02

**Authors:** Yun Shi, Min Shen, Yong Gu, Yang Chen, Kuanfeng Xu, Ji Hu, Chen Fang, Heming Guo, Ning Xu, Guofeng Wang, Weiping Lu, Sha Tao, Songqing Zhao, Chengxia Fang, Jianhua Ma, Rengna Yan, Rui Sun, Li Qian, Chenguang Wu, Hui Jiang, Tao Yang

**Affiliations:** 1 Department of Endocrinology and Metabolism, The First Affiliated Hospital with Nanjing Medical University, Nanjing, China; 2 the Second Affiliated Hospital of Soochow University, Suzhou, China; 3 Lianyungang Clinical College of Nanjing Medical University(The Affiliated Hospital of Kangda College of Nanjing Medical University), Lianyungang, China; 4 The Affiliated Huai’an No. 1 People’s Hospital of Nanjing Medical University, Nanjing, China; 5 Yangpu Hospital, Tongji University, Shanghai, China; 6 Department of Endocrinology, Nanjing First Hospital, Nanjing Medical University, Nanjing, China; 7 Sir Run Run Hospital, Nanjing Medical University, Nanjing, China; 8 Endocrinology Department, Affiliated People’s Hospital, Jiangsu University, Zhenjiang, China

**Keywords:** Type 1 diabetes, Type 1 diabetes

## Abstract

**Background and Aims:**

We aimed to explore whether saxagliptin, a dipeptidyl peptidase-IV inhibitor, could ameliorate glucose fluctuations and maintain β-cell function in T1DM.

**Methods and Results:**

A multicentre, open-label, randomised trial was performed, including 184 T1DM patients from six medical centres. These patients received insulin with or without saxagliptin at 5 mg per day for 24 weeks. The primary endpoint was the change from the baseline value of the MAGE, as measured by a CGMS after 24 weeks. The secondary endpoints included the change from baseline value of islet function during the 3-hour BMTT, HbA1c, and insulin dosage. The exploratory analysis was the influence of SNPs in the incretin-related genes on saxagliptin treatment outcomes. No differences were observed between the two groups in MAGE after treatment for 24 weeks. The change of C-peptide_max_ levels from baseline to 24 weeks in SAXA group (insulin plus saxagliptin) was higher than in CONT group (insulin only) [*p* = 0.040]. No difference were observed between the groups in HbA1c, insulin dosage after 24 weeks. In SAXA group, rs10305439, rs10305441 of GLP1R and rs6233 of PCSK1/3 were associated with HbA1c response (*p* = 0.026, 0.019, and 0.048 respectively); the G allele of rs2143734 of GLP1R were associated with lower change of fasting C-peptide from baseline (*p* = 0.029)

**Conclusions:**

The saxagliptin did not ameliorate glucose fluctuations; however, it appeared to maintain β-cell function to some extent, and SNPs in the incretin-related gene may indicate responsiveness to DPP-IV inhibitors in T1DM.

**ClinicalTrials.** Gov number, NCT 02307695

## Introduction

Type 1 diabetes mellitus (T1DM) is characterised by immune-mediated β-cell destruction, resulting in as little as 10–20% residual functional tissue at the time of diagnosis [1]. The prevalence of T1DM is 0.02% among people aged 0–14 years, with a 3% annual increase in incidence [2]. Studies have shown that the frequency of inadequate glycaemic control in T1DM is approximately 50% [3]. A significant association exists between glycaemic fluctuations and the incidence of microvascular and macrovascular complications in diabetes [4].

Insulin remains a cornerstone in the treatment of individuals with T1DM. Nonetheless, insulin therapy insulin therapy falls short of accurately replicating the physiological regulation of insulin secretion by pancreatic β-cells. This limitation poses significant challenges in achieving and maintaining stringent tight glycemic control, particularly over the long term [5]. In T1DM patients, their insulin secretion after meal is not enough to suppress glucagon, which leads to postprandial hyperglucagonemia. A previous study showed that glucagon secretion in T1DM patients increased by 37% over 12 months in response to a physiological stimulus (mixed meal) while C-peptide secretion declined by 45% after 1 year of diagnosis [6]. However, basal hyperglucagonemia may contribute to impaired glucagon regulation in T1DM [7]. Therefore, adjunctive therapies that target glucagon may be advantageous for T1DM.

Dipeptidyl peptidase-IV (DPP-IV) inhibitors are a class of oral hypoglycaemic agents that enhance endogenous glucagon-like peptide 1 (GLP-1) activity by reducing GLP-1 degradation, which augments insulin secretion and decreases glucagon release. Saxagliptin is a highly potent, selective, reversible DPP-IV inhibitor, which is commonly used to treat type 2 diabetes mellitus (T2DM). DPP-IV inhibitors reduce glycaemic variability and improve β-cell function in patients with T2DM [8], suggesting that these agents may also be valuable treatment options for patients with T1DM. Furthermore, the administration of DPP-IV inhibitors to non-obese diabetic (NOD) mice preserved β-cell mass and stimulated β-cell replication, decreasing the inflammatory profile in the pancreatic microenvironment and increasing the frequency of systemic regulatory T cells [9, 10]. Although some clinical studies have examined the effects of DPP-IV inhibitors in T1DM, these studies have reached inconsistent conclusions [11–13]. The inconsistencies observed may be attributed to factors such as limited sample sizes, insufficient paid to blood glucose fluctuations,and patient heterogeneity, including variations in islet function and single nucleotide polymorphisms (SNPs) within the incretin-related genes.

The incretin hormones, GLP-1 and glucose-dependent insulinotropic peptide (GIP), enhance glucose -stimulated insulin secretion from pancreatic β-cells and contribute to β-cell differentiation and growth through structurally related G protein-coupled receptors, the GIP receptor (GIPR) and GLP-1 receptor (GLP1R). However, they are rapidly degraded by the enzyme DPP IV. GLP-1 is post-translationally cleaved from the product of the proglucagon gene (GCG) by prohormone convertase (PC) enzymes 1/3 (PCSK1/3). Therefore, we wanted to further analyze the influence of SNPs in the incretin-related genes (GCG, GLP1R, DPP4, PCSK1, GIP and GIP1R) on the treatment effects of DPP-IV inhibitors.

Thus, the aim of this study was to investigate the impact of a 24-week regimen of saxagliptin (5 mg daily) treatment added to insulin therapy on glycemic control in heterogeneous T1DM patients (including variations in β-cell function stratification and SNPs within the incretin-related genes).

## Materials and methods

### Study design

This study was designed as a randomised, open-label study to evaluate the efficacy and safety of saxagliptin in combination with insulin therapy in T1DM patients from 6 centres. Randomisation was done by random sequence generation and was stratified by centre. A total of 184 subjects, between 12 and 65 years, from inpatient wards and hospital-affiliated clinics were enrolled in this study: 80 patients from the first affiliated hospital with nanjing medical university, 28 patients from the second affiliated hospital of soochow university, 28 patients from Lianyungang clinical college of Nanjing medical university (the affiliated hospital of kangda college of Nanjing medical university), 20 patients from the affiliated Huai’an No. 1 people’s hospital of Nanjing medical university, 16 patients from Nanjing first hospital and 12 patients from affiliated people’s hospital of Jiangsu university. The study included a 3-week enrolment period (period A) and a 24-week treatment period (period B). After the enrolment period, patients were randomly assigned to one of two groups for 24-weeks: insulin therapy with or without saxagliptin (SAXA group and CONT group). Saxagliptin treatment was initiated and maintained at 5 mg saxagliptin every morning until the completion of the study. Compliance with study drug administration was assessed by patient diaries, drug accountability, and study drug records. Compliance with drug treatment was larger than 95% in all study participants.

Treatment period spans 24 weeks, during which there are 3 site visits and 7 telephone visits which will help the patients adjust their insulin dose to avoid hypoglycaemia. Insulin dosage adjustment method was as below: FPG < 4.4 mmol/L, adjustment of -2U insulin; FPG 4.4–6.1 mmol/L, adjustment of 0U insulin; FPG6.2–7.8 mmol/L, adjustment of +2U insulin; FPG7.9-10.0 mmol/L, adjustment of +4U insulin; FPG > 10 mmol/L, adjustment of +6U insulin; PPG 2 h < 8 mmol/L, adjustment of 0U insulin; PPG 2 h 8–10 mmol/L, adjustment of +2U insulin; PPG 2 h 10–15 mmol/L, adjustment of +2-4U insulin; PPG 2 h > 15 mmol/L, adjustment of +4-6U insulin;

### Procedures

**Fig. 1:**
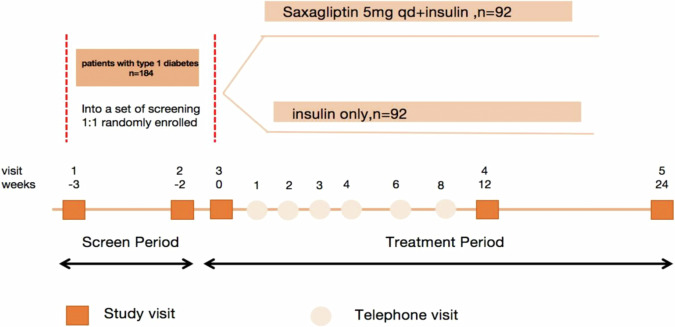
Study plan and procedures. In addition to visits at weeks –3 and –2 (randomisation) during the screening period, study visits occurred at weeks 0, and at weeks 12, 24. Telephone visits occurred at weeks 1, 2, 3, 4, 6 and 8.

In addition to visits at weeks –3 and –2 (randomisation) during the screening period, study visits in the hospital occurred at weeks 0, 12 and 24. Telephone visits occurred at weeks 1, 2, 3, 4, 6 and 8. Insulin dose, frequency of hypoglycaemic events were collected. Glucose concentrations were measured using a continuous glucose monitoring system (CGMS) at week -2 and 24. HbA1c levels were assessed at weeks −3, 12 and 24; daily insulin doses were recorded at weeks −2, 12, and 24. A steamed bread meal tolerance test (BMTT) was conducted under fasting state without bolus insulin injection at weeks −2, 12 and 24. The whole-blood samples were collected from the participant for SNPs genotyping at weeks 0.(Fig. 1)

### Participants

T1DM patients aged 12 to 65 years were eligible for participation in the trial if they met the following inclusion criteria: HbA1c level of 6.5–10.0%, and positivity for at least one of the islet autoantibodies [insulinoma-associated protein 2 autoantibody (IA2A), glutamic acid decarboxylase 65 autoantibody (GADA), or zinc transporter 8 autoantibody (ZnT8A)]. The diagnosis was based on the criteria established by the World Health Organization (WHO) and the American Diabetes Association (ADA) [14]. Patients could be treated with either multiple daily injections (MDI) or continuous subcutaneous insulin infusion (CSII). Patients were excluded from the study if they met any of the following criteria: diagnosis as type 2 diabetes; presence of unstable acute or chronic diabetic complications requiring hospitalization; oral administration of any medication affecting insulin sensitivity within the previous 3 months; diagnosis of chronic pancreatitis or other pancreatic disorders; presence of severe active infections or significant impairment of immune response; diagnosis of any other autoimmune diseases; pregnancy, lactation or inability to exclude the possibility of pregnancy in women; participation in any other drug trial within 3 months prior to enrollment; impaired liver or kidney function, defined as ALT/AST ≥ 3 times the upper limit of normal levels or creatinine clearance(CrCl) ≤ 50 ml/min; concomitant use of potent CYP3A4/5 inhibitors; history of multiple drug allergies; presence of allergic diseases associated with high sensitivity and drug-induced onset or inability to understand or sign the informed consent form.

The institutional review board at each trial centre approved the protocol and the consent form (2014-SR-123). All participants provided written informed consent. We conformed with the Helsinki Declaration of 1975 (as revised in 2008) concerning Human and Animal Rights, and followed out the policy concerning Informed Consent as shown on Springer.com.

This trial is registered at ClinicalTrials.gov, NCT02307695.

### Endpoints

The primary endpoint was the change from baseline in the mean amplitude of glycaemic excursions (MAGE) at week 24, which was assessed by CGMS. Secondary endpoints included the change of islet function from baseline during the 3-hour BMTT, HbA1c levels, and insulin dosage. The exploratory analysis was the influence of SNPs in the incretin-related genes (genetic variants in GCG, GLP1R, DPP4, PCSK1, GIP and GIP1R) on saxagliptin treatment outcomes.

### Clinical and biochemical assessments

Venous blood samples were collected in the morning after overnight fasting for biochemical parameter analysis. A Beckman AU5800 automated analyser (Beckman Coulter, Miami, FL) was used to measure biochemical levels and plasma glucose. HbA1c levels were measured by the ARIANT II haemoglobin testing system (Bio-Rad, Munich, Germany). After 12 h of fasting, patients were given a BMTT without insulin injection or hypoglycaemic drugs. Serum samples were assayed for C-peptide levels before and 30, 60, 120, and 180 min after the meal. The AUC_C-peptide_ was calculated according to the trapezoid method. C-peptide_max_ was defined as the highest C-peptide level during each BMTT and ΔCP_120_ was defined as C-peptide at 120 min minus C-peptid at 0 min (ΔCP_120_ = 2hCP-FCP) and ΔCP_180_ was defined as C-peptide at 180 min minus C-peptid at 0 min(ΔCP_180_ = 3hCP-FCP) [15]. Serum levels of C-peptide were analysed by an automatic electrochemical luminous instrument (Roche, Switzerland). All the parameters of continuous glucose monitoring were calculated from each CGMS output, which was extracted using the CGMS 3.0 software package [Medtronic MiniMed, MMT-7310 version 3.0 C (3.0.128)]. The duration of the CGMS wear is 3 days to calculate glucose levels.

### Genotyping assay

Genomic DNA extracted from peripheral blood lymphocytes was genotyped using the Illumina Human OmniZhongHua-8 platform, providing 787,224 qualified SNPs. Then we extracted the incretin-related genes (GCG, GLP1R, DPP4, PCSK1, GIP and GIP1R) SNPs from it based on quality control and imputation protocols [16]. Detailed information on these SNPs is presented in Supplementary Table S1. Genotyping was conducted for 50 patients in SAXA group and 50 patients in CONT group.

### Statistical analysis

An independent statistician performed all statistical analyses. All data were entered into SPSS 17.0 software (SPSS Inc., Chicago, IL). Continuous data are presented as the mean ± standard deviation or the median with interquartile range (IQR), and categorical variables are presented as the number and percentage. We compared two groups using the t-test or the non-parametric Mann–Whitney U test for continuous variables and the Chi-square test for categorical variables. The changes from baseline for primary and secondary endpoints were analysed using a covariance pattern model, with treatment as the classification variable and the baseline value as a covariate. Patients were divided into ‘response group’ and ‘non-response group’ according to whether the change of examination indicators from baseline indicators was greater than or less than 0 after 24 weeks of treatment. Multivariate logistic regression analyses were also performed to assess SNPs effects on saxagliptin treatment outcomes. Differences in categorical variables were analysed using the Cochran–Mantel–Haenszel (CMH)/Chi-square test. *P* < 0.05 was considered significant.

The primary, secondary, analyses were based on the full analysis set (FAS) and included all randomised patients. We compared the treatment groups according to the randomised treatment, regardless of whether the treatment was actually received or whether any protocol deviations or violations were reported. The incidence of adverse events (AEs) was summarised. Analyses for safety and tolerability endpoints were summarised using descriptive statistics for continuous variables or frequency counts and percentages for categorical variables.

With a total of 184 patients randomized and treated (*n* = 92 per group), there would be 80% power to detect a 1.0 mmol/L difference between the 2 randomized treatment groups in absolute change from baseline to Week 24 in MAGE at the 5% level, assuming a standard deviation of change from baseline MAGE of 2.23%, dropout rate=15%.

## Results

**Fig. 2:**
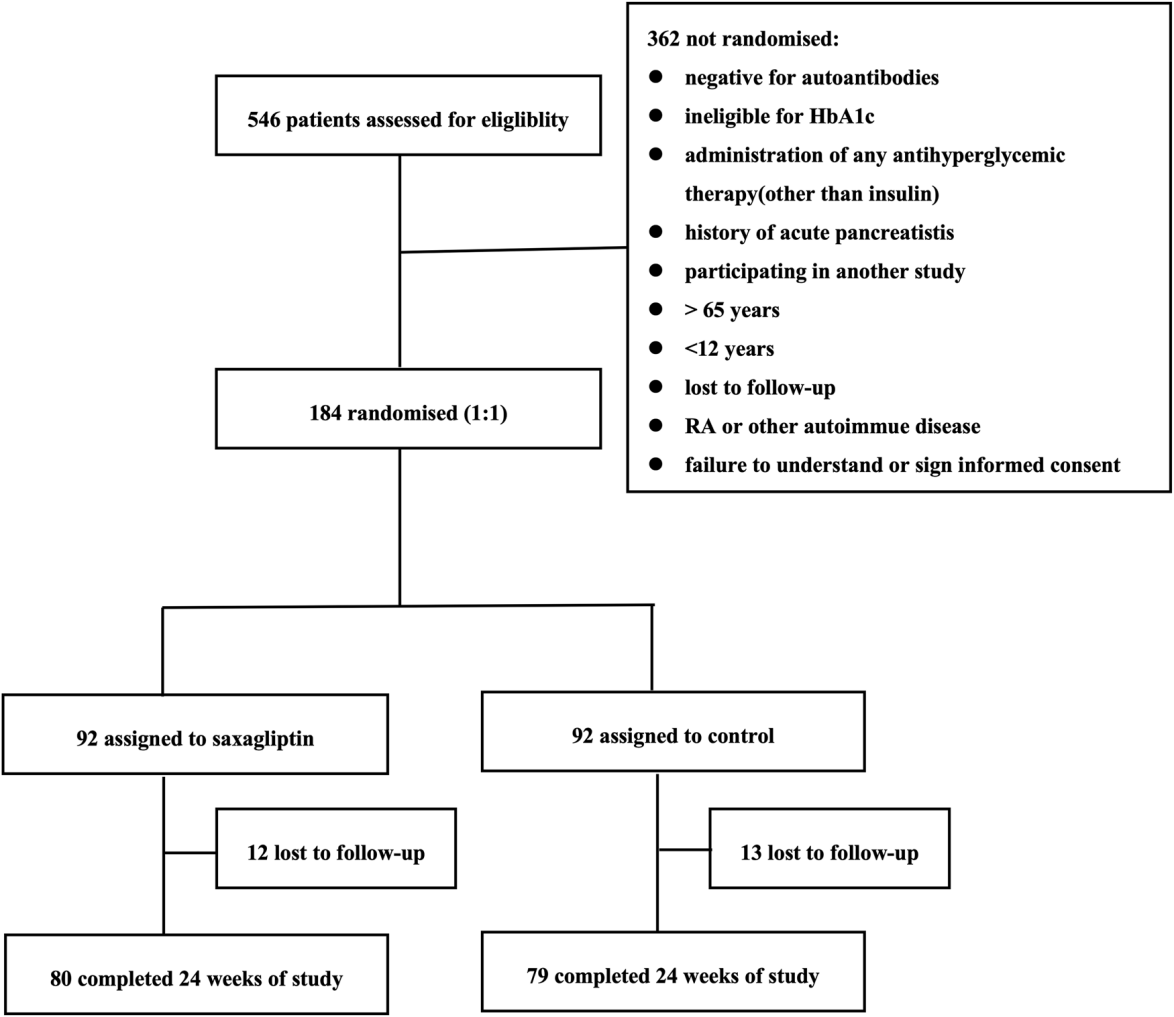
Trial profile of this study. The trial profile summarizing the flow of patients through the trail from the pre-randomization recruitment period to the post-randomization follow-up and analysis.

A total of 546 patients from 6 hospitals were assessed for eligibility in this study between 16 December 2014 and 28 July 2017. Of these, 184 patients were randomly assigned in a 1:1 ratio to one of two study groups: SAXA group (insulin plus saxagliptin 5 mg, *n* = 92) vs CONT group (insulin only, *n* = 92), as illustrated in Fig. 2. Among these, 159 (86%) randomly assigned patients completed the 24-week treatment period. The predominant reason for study non-completion was loss to follow-up.

### Subject characteristics

The mean age of patients in SAXA group was 35.6 ± 13.4 years vs 33.1 ± 13.6 years in CONT group (*p* = 0.203). SAXA group included 50 (54.35%) male subjects compared with 47 (51.09%) male subjects in CONT group (*p* = 0.714). The mean ages of T1DM onset were 29.98 ± 13.86 years in SAXA group and 26.91 ± 15.00 years in CONT group (*p* = 0.163). 12.5% (23/184) adolescence patients were enrolled and randomly assigned to a study [SAXA group (*N* = 9) vs CONT group (*N* = 14)]). Patients reported a median (IQR) disease duration of 3.26 (1.28, 7.57) years in SAXA group and 3.84 (2.02, 8.33) years in CONT group (*p* = 0.371). No significant differences between the two groups were observed for height, body weight, body mass index (BMI), systolic blood pressure (SBP), or diastolic blood pressure (DBP). The two groups had similar HbA1c, fasting C-peptide (FCP), C-peptide at 180 min and AUC_C-peptide180_ levels at baseline. There was no significant difference in the total daily insulin dose between the two groups. Biochemical variables and islet autoantibody positivity rates were not significantly different between groups. Overall, the baseline characteristics and demographics were comparable between the two groups (Table 1).

**Table 1:** Demographic and baseline characteristics.

	SAXA group (Saxaglitpin + insulin) (N = 92)	CONT group (Insulin) (*N* = 92)	p*-value*
Age (y)	35.6 ± 13.4	33.1 ± 13.6	0.203
Age of onset (y)	29.98 ± 13.86	26.91 ± 15.00	0.163
Diabetic duration (y)	3.26 (1.28, 7.57)	3.84 (2.02, 8.33)	0.371
Sex (male%)	54.35%	51.09%	0.714
Height (cm)	167.29 ± 8.15	166.02 ± 8.03	0.294
Weight (kg)	60.63 ± 10.13	58.58 ± 9.18	0.159
BMI (kg/m^2^)	21.57 ± 2.50	21.21 ± 2.59	0.344
SBP (mmHg)	116.80 ± 12.17	116.61 ± 11.88	0.870
DBP (mmHg)	73.34 ± 8.88	75.05 ± 9.67	0.487
HbA1c (%)	8.16 ± 1.09	8.08 ± 1.01	0.590
Insulin dose (U/kg/d)	0.65 ± 0.22	0.65 ± 0.20	0.834
FCP (pmol/L)	90.10 (3.33,116.55)	75.38 (3.33, 112.69)	0.391
C-peptide_180_ (pmol/L)	78.92(3.33, 309.7)	125.87(3.33, 303.05)	0.879
AUC_C-peptide180_ (pmol/L·h)	160.67(9.99, 715.63)	296.45(9.99, 757.20)	0.973
Insulin injection			0.805
MDI	91.76%	90.70%	
CSII	8.24%	9.30%	
ZnT8A (%)	25.93%	33.33%	0.298
IA2A (%)	44.44%	55.56%	0.182
GADA (%)	85.19%	85.00%	0.970

Data are presented as the *n* (%), the mean ± SD, or the mean interval (25, 75); *MDI* multiple daily injections, *CSII* continuous subcutaneous insulin infusion, *BMI* Body mass index, *DBP* diastolic blood pressure, *FCP* fasting C-peptide, *HbA1c* glycated haemoglobin A1c, *SBP* systolic blood pressure; *ZnT8A* zinc transporter 8 autoantibody, *IA2A* protein tyrosine phosphatase 2 autoantibody, *GADA* glutamic acid decarboxylase autoantibodies, *AUC* area under the curve. The AUC_C-peptide_ was calculated according to the trapezoid method. All *p* values were two-tailed and *p* < 0.05 was been considered as significant.

### Glucose levels by continuous glucose monitoring system (CGMS)

No significant changes in mean daily glucose levels were observed after 24 weeks of treatment in either SAXA group or CONT group (*p* = 0.502; Table 2). In addition, SAXA group or CONT group exhibited similar percentages of time spent in the target glycaemic range (3.9–11.1 mmol/L), hyperglycaemic range (>11.1 mmol/L), and hypoglycaemic range (<3.9 mmol/L). Meanwhile, the variables measured by the CGMS, including MAGE, standard deviation (SD), coefficient of variation (CV), high blood glucose index (HBGI), and low blood glucose index (LBGI), were comparable between the two groups. Furthermore, no significant associations between SNPs and MAGE, SD, CV, LBGI, HBGI response of 24 weeks treatment of saxagliptin were observed in SAXA group.

**Table 2:** Change in continuous glucose monitoring system (CGMS) values after 24 weeks treatment.

	SAXA group			CONT group			*p-value*
	Baseline (n = 74)	24 weeks (*n* = 57)	Change from baseline	Baseline (n = 69)	24 weeks (*n* = 57)	Change from baseline	
CGM mean daily glucose (mmol/L)							
	9.01 ± 2.25	8.81 ± 2.04	−0.35	9.01 ± 2.37	9.07 ± 2.62	−0.03	0.502
CGM% time in ranges (mmol/L)							
<3.9	4.17 ± 7.18	4.82 ± 7.5	0.84	5.62 ± 11.86	6.03 ± 9.21	−1.25	0.942
3.9–7.8	38.95 ± 20.95	39.90 ± 20.31	1.31	40.34 ± 22.76	40.39 ± 22.84	1.21	0.983
>7.8	56.88 ± 23.2	54.99 ± 23.59	−2.8	54.04 ± 25.36	53.58 ± 25.37	0.04	0.605
>11.1	26.7 ± 22.43	22.27 ± 20.73	−6.78	25.99 ± 23.02	27.29 ± 21.65	−1.06	0.239
CGM variability measures							
MAGE	6.67 ± 2.88	6.48 ± 3.10	−0.10	6.61 ± 2.61	6.45 ± 2.89	−0.16	0.660
SD	3.00 ± 2.95	2.68 ± 1.12	−0.02	2.82 ± 1.01	2.86 ± 1.10	−0.04	0.956
CV	4.23 ± 3.12	4.09 ± 2.46	0.27	3.66 ± 1.84	3.63 ± 1.55	0.15	0.838
HBGI	10.26 ± 6.8	9.27 ± 5.9	−1.29	10.46 ± 6.65	9.73 ± 7.36	−1.51	0.880
LBGI	3.02 ± 3.4	3.03 ± 2.79	0.00	2.91 ± 3.49	4.11 ± 4.33	0.86	0.326

Data are presented as the mean ± SD; *CGM* continuous glucose monitoring, *CGMS* continuous glucose monitoring system, *CV* coefficient of variation, *HBGI* high blood glucose index, *LBGI* low blood glucose index, *MAGE* mean amplitude of glucose excursions, *SD* standard deviation. All p values were two-tailed and p < 0.05 was been considered as significant.

Since blood glucose fluctuations are closely linked to β-cell function, we analyzed CGMS data by categorizing patients based on different β-cell function. Patients with varying β-cell function randomised into different treatment groups exhibited a non-significant trend indicating a reduction in MAGE (Table 3).

**Table 3:** Change in mean amplitude of glucose excursions (MAGE) values stratificated by β-cell function after 24 weeks treatment.

	SAXA group			CONT group			
FCP	Baseline (n = 66)	24 weeks (*n* = 60)	Change from baseline	Baseline (*n* = 60)	24 weeks (*n* = 56)	Change from baseline	*p-value*
≤3.33 pmol/L	7.54 ± 2.67	6.80 ± 2.44	−0.74 ± 2.20	6.45 ± 2.84	6.45 ± 3.25	0.13 ± 3.10	0.269
3.33–200 pmol/L	6.77 ± 3.06	7.24 ± 3.53	0.57 ± 3.15	6.52 ± 2.31	7.19 ± 3.54	0.58 ± 3.17	0.603
≥200 pmol/L	4.83 ± 2.27	6.25 ± 2.97	0.24 ± 3.38	7.31 ± 2.75	5.69 ± 2.50	−1.53 ± 2.26	0.195

Data are presented as the mean ± SD; *FCP* fasting C-peptide, *MAGE* mean amplitude of glucose excursions, All *p* values were two-tailed and *p* < 0.05 was been considered as significant.

### HbA1c and insulin dosage

There was no significant difference in the change of HbA1c levels after 24 weeks relative to baseline between SAXA group [7.81 ± 1.52%] and CONT group [8.03 ± 1.40%] (*p* = 0.475). The response rate of HbA1c change was comparable between SAXA group (51.9%) and CONT group (49.2%) (*p* = 0.753). There was no significant difference in percentage change in HbA1c compared with baseline after 24 weeks either[-3.93(-12.08, 7.04)% vs -1.49(-9.72, 6.82)%, *p* = 0.498].

No significant difference in the change in insulin dosage after 24 weeks of treatment relative to baseline was observed between SAXA group [0.61 ± 0.17 U/kg/d] and CONT group [0.63 ± 0.22 U/kg/d] (*p* = 0.909).

After stratifying patients according to different β-cell function, no difference was found in changes HbA1c and insulin dosage between the two groups either.

### β-cell function

**Fig. 3:**
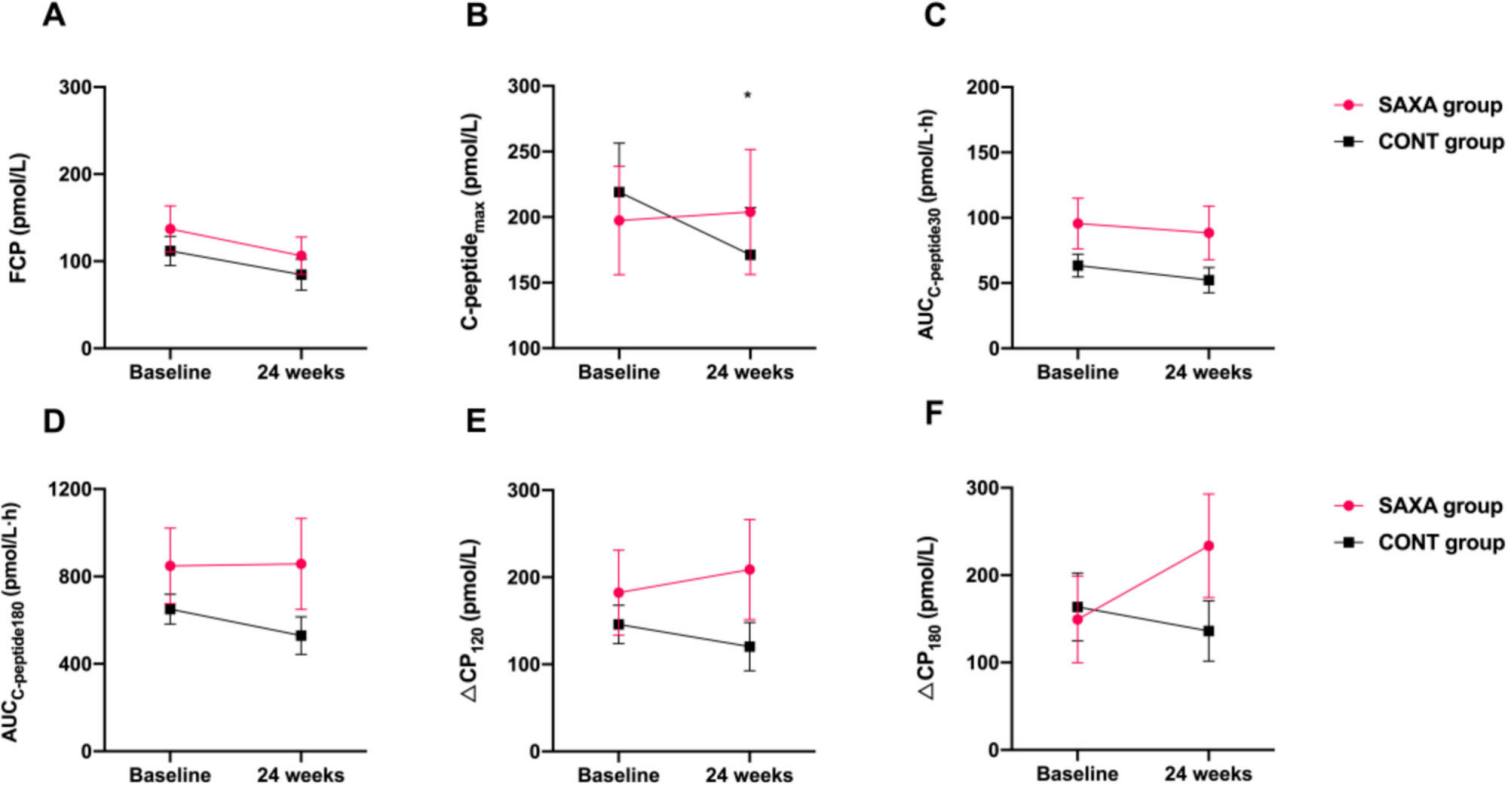
Change in β-cell function values after 24 weeks treatment in patients with remaining β-cell function. The change of fasting C-peptide(**A**), C-peptide_max_(**B**), AUC_C-peptide30_(**C**), AUC_C-peptide180_(**D**), ΔCP_120_ (**E**) and ΔCP_180_ (**F**) from baseline to 24 weeks in SAXA group and CONT group. All *p* values were two-tailed and *p* < 0.05 was been considered as significant. *p < 0.05.

92.39% (170/184) patients assessed FCP levels (*N* = 86 in SAXA group vs *N* = 84 in CONT group) and 75.54% (139/184) patients performed BMTT (*N* = 75 in SAXA group vs *N* = 64 in CONT group) at baseline. Islet function assessment after 24 weeks follow-up were shown in Table 4. No significant difference in the change from baseline FCP levels [-11.66 (-42.29, 0.00) vs -21.74 (-53.28, 3.33), *p* = 0.691] and AUC_C-peptide180_ [0.00 (-69.85, 36.05) vs -58.57 (-188.64, 0.05) pmol/L·h, *p* = 0.168)] was observed after 24 weeks between SAXA group and CONT group. The change of C-peptide_max_ levels from baseline to 24 weeks in SAXA group was higher than in CONT group [0.00 (-38.96, 7.16) vs -0.03 (-106.56, 5.66) pmol/L, *p* = 0.04]. Analyses also performed in patients with remaining β-cell function (fasting C peptide > 3.33 mmol/L), and similar conclusions were obtained (Fig. 3).

**Table 4:** Change in β-cell function values after 24 weeks treatment.

	SAXA group			CONT group			
	Baseline	24 weeks	Change from baseline	Baseline	24 weeks	Change from baseline	*p-value*
FCP (pmol/L)	90.10(3.33, 116.55)	86.45(3.33, 104.96)	-11.66(-42.29, 0.00	75.38(3.33, 112.69)	61.93 (3.33, 83.25)	21.74(-53.28, 3.33)	0.691
C-peptide_max_ (pmol/L)	79.90 (3.33, 353.00)	55.28(3.33, 362.97)	0.00(-38.96, 7.16)	136.84(3.33, 329.60)	118.22(9.82, 234.77)	-0.03(-106.56, 5.66)	0.040*
AUC_C-peptide30_ (pmol/L·h)	16.25(1.70, 77.85)	6.25(1.70, 67.40)	-0.46(-15.28, 0.00)	24.10(1.70, 58.70)	22.80(3.05, 60.35)	-0.50(-18.90, 1.50)	0.814
AUC_C-peptide180_ (pmol/L·h)	160.67(9.99, 715.63)	89.58(9.99, 650.18)	0.00(-69.85, 36.05)	296.45(9.99, 757.20)	275.81(27.31, 574.43)	-58.57(-188.64, 0.05)	0.168
ΔCP_120_ (pmol/L)	26.64(0.00, 193.04)	13.30(0.00, 169.80)	0.00(-23.31, 0.00)	49.06(0.00, 153.18)	53.30(0.00, 114.60)	0.00(-86.58, 14.65)	0.925
ΔCP_180_ (pmol/L)	26.64(0.00, 174.50)	24.98(0.00, 143.52)	0.00(-26.64, 27.98)	43.46(0.00, 186.48)	62.11(0.165, 148.52)	0.00(-83.25, 17.73)	0.305

Data are presented as the mean interval (25, 75). *FCP* Fasting C-peptide. All *p* values were two-tailed and *p* < 0.05 was been considered as significant. *p < 0.05.

### Effects of DPP4 associated SNPs on saxagliptin treatment

**Fig. 4:**
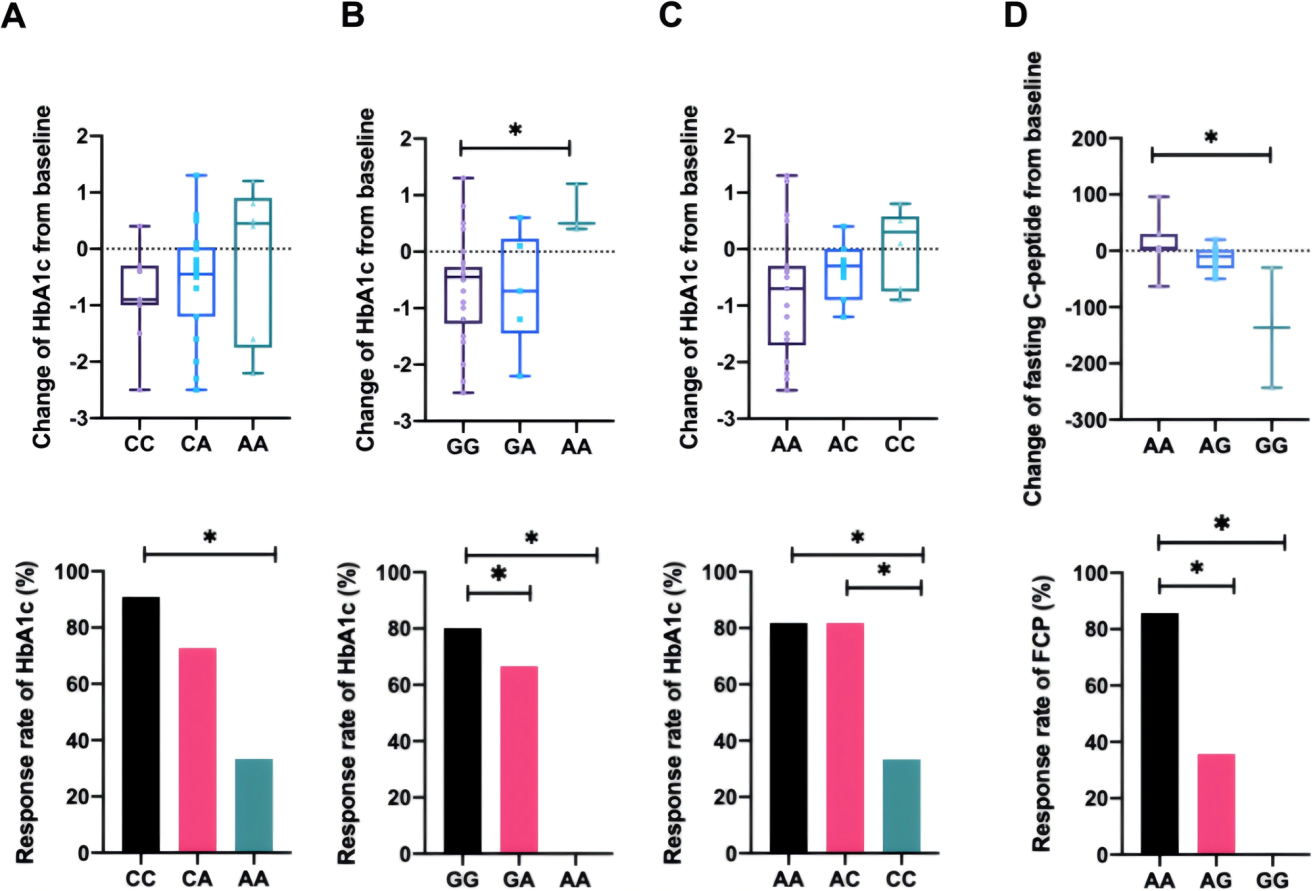
Change of HbA1c and FCP in patients with different genotypes in SAXA group after 24 weeks treatment. The upper part shows the change value of HbA1c or fasting C-peptide in patients with different genotypes [(**A**) rs10305439, (**B**) rs10305441, (**C**) rs6233 and (**D**) rs2143734] and the bottom part shows the response rate of HbA1c or fasting C-peptide in patients with different genotypes in SAXA group. All *p* values were two-tailed and *p* < 0.05 was been considered as significant. *p < 0.05; **p < 0.01.

Genotyping was conducted for 100 patients (50 in SAXA group and 50 in CONT group). We aimed to investigate the association of SNPs in the incretin-related genes (GCG, GLP1R, DPP4, PCSK1, GIP and GIP1R) on the treatment effects of saxagliptin. Interestingly, we found in SAXA group, rs10305439 of GLP1R, rs10305441 of GLP1R and rs6233 of PCSK1 were associated with HbA1c response (*p* = 0.026, 0.019, and 0.048 respectively). Patients with mutation allele of these variants in SAXA group had lower response rate of HbA1c (Fig. 4A-C). At the same time, we found in SAXA group the G allele of rs2143734 of GLP1R were associated with lower change of FCP from baseline (*p* = 0.029) (Fig. 4D). Meanwhile, there were no SNPs associated with response rate of HbA1c and FCP in CONT group. No significant associations between SNPs and CGMS data, insulin dose, C-peptide_max_, AUC_C-peptide30_, AUC_C-peptide180_, ΔCP_120_, ΔCP_180_ response of 24 weeks treatment were observed in SAXA group.

### Safety evaluation

No clinical chemistry changes in value relative to baseline were observed over time in any group (Table S2). The proportions of patients who experienced at least one AE were similar between groups. A serious AE (myocardial infarction) was reported in one patient without saxagliptin. Saxagliptin was well tolerated by Chinese patients with T1DM.

## Discussion

In this study, we evaluated the use of the DPP-IV inhibitor saxagliptin as an adjunct to insulin therapy for patients with T1DM in a multicentre, open-label, randomised trial over 24 weeks. This is the first study of saxagliptin among Chinese T1DM patients. In the present study, these results indicate that saxagliptin added to insulin did not improve glycaemic fluctuations in patients with T1DM. However, saxagliptin appeared to maintain β-cell function in T1DM patients receiving insulin treatment to some extent. Importantly, this is the first attempt to investigate the association of SNPs in the incretin-related genes (GCG, GLP1R, DPP4, PCSK1, GIP and GIP1R) on the treatment effects of DPPV inhibitors. SNPs in the incretin-related gene may indicate responsiveness to DPP-IV inhibitors in T1DM.

Numerous small-scale studies have investigated the clinical efficacy of DPP-IV inhibitors in glucose control and variability among adult patients with T1DM. However, these studies have yielded inconsistent results regarding the outcomes of DPP-IV inhibitor co-therapy in T1DM. Farngren et al. demonstrated a-28 days regimen of the DPP-IV inhibitor vildagliptin in T1DM had a small benefit on HbA1c reduction [13]. Ellis et al. reported that 4 weeks of sitagliptin adjunct therapy in T1DM significantly improved HbA1c levels (−2.91 ± 1.16 mmol/l) [17]. By contrast, George et al. reported that saxagliptin co-therapy did not reduce glucose variability, hypoglycaemia frequency or awareness, and did not improve counterregulatory hormonal responses during experimental hypoglycaemia in individuals with established C-peptide negative T1DM [18]. Garg et al. conducted a 20-week, double-blind study that randomised 125 patients to receive either 100 mg/day sitagliptin or matching placebo in a 1:1 fashion. Sitagliptin use in patients with T1DM did not change glucagon AUC values, HbA1c levels, insulin dose, or weight, despite a post-meal rise in GLP-1 levels. Among patients who had detectable C-peptide levels, non-significant trends towards greater reductions in mean glucose and time spent in the hyperglycaemic range were observed for the sitagliptin group [19]. Furthermore, Guo et al. preformed a meta-analysis in which six randomized controlled trials with a total of 228 individuals were finally included and found that DPP-IV inhibitors reduced daily insulin dosage significantly but did not reduce HbA1c level [20]. We inferred that small sample numbers, little attention paid to blood glucose fluctuations, and heterogeneity among patients (including, islet function and SNPs in the incretin-related genes) may contribute to these inconsistencies.

On the one hand, it has been inferred that fluctuations in blood glucose are closely related to β-cell function, leading to the hypothesis that whether the clinical efficacy of DPP-IV inhibitor on glucose control and variability may be affected by islet functional levels in adult T1DM patients in previous studies? To explore this, we conducted an analysis among individuals with different islet functional levels. Patients with different β-cell function randomised into different treatment groups, revealing a non-significant trend indicating a reduction in MAGE. On the other hand, our study, using both CGMS and HbA1c to evaluate the mean glucose level and glucose fluctuations. In the present study, no changes were observed in the CGMS data and this study did not show a reduction in HbA1c levels, our secondary endpoint, and no significant difference was observed between the two groups. Interestingly, we found in SAXA group, rs10305439 and rs10305441 of GLP1R and rs6233 of PCSK1 were associated with HbA1c response. Patients with mutation allele of these variants had lower response rate of HbA1c. These results suggest that saxagliptin may be better able to modulate blood glucose homeostasis in specific patients, further investigation remains necessary.

Many studies have indicated a beneficial effect of DPP-IV inhibition on β-cell mass in animal models of T1DM. Kim et al. demonstrated that sitagliptin could reduce the incidence of diabetes in islet-transplanted NOD mice, whlie increased β-cell area and decreased insulinitis [21]. Cho et al. demonstrated that long-term administration of the DPP-IV inhibitor increased the mass, replication and neogenesis of β-cell in streptozotocin-treated mice [22]. Jacob et al.reported that DPP-IV inhibition is able to reduce the incidence of diabetes and increase the β-cell mass in female NOD mice [9]. However the efficacy of saxagliptin to protect beta-cell function in T1DM patients have not been well explored. The pathogenesis of T1DM in human is more complex; more population studies are needed to verify the results of animal experiments. A randomized controlled clinical trial conducted in China and published in 2021 showed that sitagliptin combined with insulin therapy appeared to delay the decline of islet β-cell function in patients with latent autoimmune diabetes in adults (LADA) to some extent [15]. However, a meta-analysis has been undertaken by Wu et al. to investigate the effects of incretin-based therapies (DPP-IV inhibitors or GLP-1R agonists) on β-cell function in T1DM patients and these therapies did not preserve β-cell function in patients with T1DM patients [23].

FCP is frequently used in clinical practice due to its good correlation with BMTT; the C-peptide_max_, AUC_C-peptide_, ΔCP_120_, ΔCP_180_ values during BMTT may reflect the secretory capacity in response to a mixed meal. Our study also did not investigate the improvement effects of saxagliptin in the FCP and AUC_C-peptide_ after 24 weeks. However, we found in SAXA group (insulin plus saxagliptin) the G allele of rs2143734 of GLP1R were associated with lower change of FCP from baseline; the change of C-peptide_max_ levels from baseline to 24 week in SAXA group was higher than in CONT group, which demonstrated the protective benefit of saxagliptin on β-cell function in T1DM patients to some extent.

There are several strengths in the present study. Firstly, our study used a robust randomized controlled trial design; compared to previous studies with small sample size or short follow-up time, the present study has a large sample number and a 3-week enrolment period and a 24-week treatment period. Secondly, our study used both HbA1c and CGMS to assess glucose control and variability comprehensively; attempted to evaluate β-cell function by the combined application of several methods, such as FCP, C-peptide_max_, ΔCP_120_, ΔCP_180_ and AUC_C-peptide_ in BMTT among most patients, then analysed the clinical efficacy in patients stratified by islet function; enrolled patients as well as 12.5% adolescent, about one third patients with poor islet function when enrolled; which makes our study more credible. Thirdly, this is the first attempt to investigate the association of SNPs in the incretin related gene with the clinical efficacy in T1DM patients. Importantly, inter-individual variance in the responsiveness to DPP-IV inhibitors were reported [24–26]. We asked whether genetic variation in the incretin related gene affects in C-peptide secretion and glucose tolerance in T1DM patients receiving DPP-IV inhibitors. In the present study, we identified SNPs mainly in the GLP1R may indicate responsiveness to DPP-IV inhibitors in T1DM patients. This gene variation may influence clinical decisions,eg., regarding the use of DPP-IV inhibitors in terms of individualized therapy for T1DM.

The limitation of our study was that we did not examine GLP-1 levels, glucagon, DPP-IV enzymatic activity, or GIP concentrations. Therefore, whether saxagliptin therapy in T1DM was ineffective at improving glucagon responses remains unclear. Additionally, SNPs in the incretin related genes were screened only in one half of T1DM patients, the sample number is too small to gain enough power to detect the effects of these variants on clinical phenotypes. Further studies with larger sample size are required to verify this association and the mechanism underlying the observed SNPs effects requires further investigation.

In conclusion, DPP-IV inhibitors may be better able to modulate blood glucose homeostasis in specific patients and appeared to maintain β-cell function to some extent. Thus, our study has implications for clinical drug selection in patients with T1DM.

## Supplementary information

Table S1 SNPs included in this study. Table S2 Safety evaluation of saxagliptin treatment.

## Data Availability

The data that support the findings of this study are available from the corresponding author upon reasonable request.
