# Supplementary material for: Heterogeneous effect of saxagliptin on glucose fluctuation and β-cell function in T1DM: a multicentre, randomised trial
Source: Nutr Diabetes. 2026 Mar 2;16:5. doi: 10.1038/s41387-026-00411-3 (PMC12987927; doi:10.1038/s41387-026-00411-3)
Supplement: Supplementary file 1 — Table S1 SNPs included in this study. Table S2 Safety evaluation of saxagliptin treatment. [file 41387_2026_411_MOESM1_ESM.docx]

**Supplementary** **Material**.

**Table S1 SNPs included in this study**

| Gene Symbol | SNPs | Genomic Position | Variant Effect | Ref | Alt |
| --- | --- | --- | --- | --- | --- |
| GCG | rs5647 | chr2:163005158 | Coding transcript intron variant | G | A |
| GLP-1R | rs2268657 | chr6:39020542 | Coding transcript intron variant | T | C |
|  | rs3799707 | chr6:39023510 | Coding transcript intron variant | T | A |
|  | rs10305432 | chr6:39023630 | Coding transcript intron variant | T | C |
|  | rs10305438 | chr6:39024448 | Coding transcript intron variant | G | A |
|  | rs10305439 | chr6:39024716 | Coding transcript intron variant | C | A |
|  | rs2143734 | chr6:39024756 | Coding transcript intron variant | A | G |
|  | rs10305441 | chr6:39024839 | Coding transcript intron variant | G | A |
|  | rs9283907 | chr6:39026703 | Coding transcript intron variant | A | G |
|  | rs2268650 | chr6:39030401 | Coding transcript intron variant | G | A |
|  | rs910170 | chr6:39032415 | Coding transcript intron variant | A | G |
|  | rs874900 | chr6:39032537 | Coding transcript intron variant | A | G |
|  | rs6923761 | chr6:39034072 | Missense variant | G | C |
|  | rs7766663 | chr6:39035782 | Coding transcript intron variant | G | T |
|  | rs12214482 | chr6:39036185 | Coding transcript intron variant | A | C |
|  | rs2235868 | chr6:39040654 | Missense variant | A | T |
|  | rs10305475 | chr6:39040701 | Synonymous variant | A | G |
|  | rs1042044 | chr6:39041502 | Missense variant | A | C |
|  | rs932443 | chr6:39042334 | Coding transcript intron variant | T | C |
|  | rs12204668 | chr6:39043796 | Coding transcript intron variant | C | A |
|  | rs2268645 | chr6:39043992 | Coding transcript intron variant | G | T |
|  | rs7769547 | chr6:39048757 | Coding transcript intron variant | A | G |
|  | rs2300613 | chr6:39049205 | Coding transcript intron variant | G | A |
|  | rs2268640 | chr6:39050384 | Coding transcript intron variant | G | A |
|  | rs2206942 | chr6:39051439 | Coding transcript intron variant | C | T |
|  | rs10305512 | chr6:39054113 | Three prime UTR exon variant | G | A |
|  | rs10305514 | chr6:39054138 | Three prime UTR exon variant | G | T |
|  | rs10305518 | chr6:39055012 | Three prime UTR exon variant | T | A |
|  | rs4714210 | chr6:39055485 | Three prime UTR exon variant | A | G |
| DPP4 | rs7565794 | chr2:162859000 | Coding transcript intron variant | T | C |
|  | rs2909447 | chr2:162864475 | Coding transcript intron variant | C | T |
|  | rs2909448 | chr2:162866255 | Coding transcript intron variant | C | T |
|  | rs2909450 | chr2:162867791 | Coding transcript intron variant | G | A |
|  | rs2268890 | chr2:162872492 | Coding transcript intron variant | A | G |
|  | rs3788980 | chr2:162878769 | Coding transcript intron variant | C | T |
|  | rs2300755 | chr2:162883962 | Coding transcript intron variant | G | A |
|  | rs2268889 | chr2:162885110 | Coding transcript intron variant | C | T |
|  | rs16822665 | chr2:162901327 | Coding transcript intron variant | C | G |
|  | rs6729733 | chr2:162907232 | Coding transcript intron variant | C | T |
|  | rs3788976 | chr2:162921135 | Coding transcript intron variant | G | A |
|  | rs1861975 | chr2:162929300 | Coding transcript intron variant | A | C |
| PCSK1 | rs6233 | chr5:95733112 | Synonymous variant | A | G |
|  | rs271923 | chr5:95737362 | Coding transcript intron variant | T | C |
|  | rs3792744 | chr5:95742313 | Coding transcript intron variant | C | A |
|  | rs156020 | chr5:95746355 | Coding transcript intron variant | T | G |
|  | rs10515237 | chr5:95751549 | Coding transcript intron variant | A | G |
|  | rs6232 | chr5:95751785 | Missense variant | T | C |
|  | rs436321 | chr5:95754483 | Coding transcript intron variant | C | A |
|  | rs6231 | chr5:95757592 | Synonymous variant | G | A |
|  | rs3792747 | chr5:95767920 | Coding transcript intron variant | T | C |
|  | rs6230 | chr5:95768847 | Five prime UTR exon variant | A | G |
| GIP | rs2291725 | chr17:47039132 | Missense variant | T | C |
|  | rs2291726 | chr17:47039254 | Coding transcript intron variant | T | C |
|  | rs8078510 | chr17:47045862 | Five prime UTR intron variant | G | A |
| GIPR | rs11671664 | chr19:46172278 | Five prime UTR intron variant | G | A |
|  | rs13306402 | chr19:46177353 | Missense variant | C | T |
|  | rs11672660 | chr19:46180184 | Coding transcript intron variant | C | T |
|  | rs12709891 | chr19:46185217 | Three prime UTR exon variant | C | A |
|  | rs2334255 | chr19:46186150 | Three prime UTR exon variant | G | T |

**Table S2 Safety evaluation of saxagliptin treatment**

|  | **SAXA group** | | | **CONT group** | | |  |
| --- | --- | --- | --- | --- | --- | --- | --- |
|  | Baseline | 24 weeks | Change from baseline | Baseline | 24 weeks | Change from baseline | *p-value* |
| Ca(mmol/L) | 2.36±0.10 | 2.36±0.10 | -0.01±0.1 | 2.36±0.11 | 2.36±0.11 | 0.01±0.12 | 0.361 |
| P(mmol/L) | 1.30±0.34 | 1.26±0.38 | 0.02±0.44 | 1.29±0.35 | 1.33±0.33 | 0.06±0.40 | 0.086 |
| Cr(umol/L) | 56.12±12.08 | 55.60±14.22 | 0.72±11.59 | 56.43±11.99 | 57.08±12.51 | 1.47±8.63 | 0.361 |
| Urea(mmol/L) | 5.07±1.27 | 5.27±1.33 | 0.08±1.18 | 5.36±1.38 | 5.40±1.26 | -0.05±1.51 | 0.590 |
| UA(umol/L) | 237.04±65.12 | 246.58±65.69 | 15.47±47.95 | 249.00±66.57 | 248.80±64.33 | 10.06±50.81 | 0.552 |
| TP(g/L) | 70.93±6.67 | 71.81±4.05 | 0.04±7.03 | 71.55±6.27 | 71.50±5.11 | 0.64±6.77 | 0.632 |
| ALB(g/L) | 43.12±4.03 | 43.55±2.90 | -0.27±4.05 | 43.33±4.15 | 43.21±3.36 | 0.17±4.46 | 0.571 |
| ALT(U/L) | 17.70±10.20 | 16.88±10.20 | -0.88±8.26 | 16.93±12.48 | 20.73±14.49 | 2.91±18.65 | 0.082 |
| AST(U/L) | 21.16±9.13 | 22.34±10.45 | 1.52±11.10 | 21.32±13.01 | 23.13±8.86 | 1.04±17.58 | 0.437 |
| LDH(U/L) | 203.43±72.71 | 235.50±176.67 | 40.05±174.16 | 213.30±84.42 | 206.67±83.91 | 3.70±106.51 | 0.739 |
| GGT(U/L) | 18.88±15.89 | 18.10±17.76 | 1.59±17.72 | 14.98±7.29 | 14.68±7.12 | 0.13±6.48 | 0.976 |
| ALP(U/L) | 105.58±122.74 | 92.11±64.01 | -19.89±130.96 | 114.89±97.14 | 100.90±69.50 | -15.21±51.30 | 0.416 |
| TG(mmol/L) | 0.86±0.43 | 0.85±0.58 | -0.01±0.49 | 0.83±0.47 | 0.80±0.39 | 0.03±0.37 | 0.250 |
| TC(mmol/L) | 4.80±0.88 | 4.71±0.90 | -0.01±0.84 | 4.74±1.09 | 4.76±0.99 | 0.08±0.83 | 0.786 |
| HDL-C(mmol/L) | 1.50±0.37 | 1.53±0.37 | -0.02±0.26 | 1.49±0.32 | 1.55±0.40 | 0.01±0.32 | 0.758 |
| LDL-C(mmol/L) | 2.98±0.67 | 2.67±0.69 | -0.23±0.61 | 2.87±0.83 | 2.82±0.63 | 0.04±0.6 | ***0.025**** |
| LP(a)(mg/L) | 237.32±250.86 | 219.50±230.67 | -5.22±90.16 | 203.90±182.99 | 218.38±210.81 | -10.17±103.72 | 0.295 |
| WBC(10^9^/L) | 5.90±1.61 | 5.72±1.46 | 0.02±1.63 | 5.87±1.44 | 5.62±1.54 | -0.05±1.59 | 0.811 |
| HGB(g/L) | 136.91±17.85 | 134.58±18.73 | -2.10±15.91 | 137.53±22.24 | 135.61±16.38 | -1.02±12.19 | 0.702 |

Data are presented as the mean ± SD; Ca, Calcium; P, phosphorus; Cr, creatinine; UA, Uric Acid; TP, total protein; ALB, albumin; ALT, alanine aminotransferase; AST, aspartateaminotransferase; LDH, lactate dehydrogenase; GGT, glutamyltransferase; ALP, alkaline phosphatase; TG, triglyceride; TC, total cholesterol; HDL-C, high density lipoprotein cholesterol; LDL-C, low density lipoprotein cholesterol; LP(a), Lipoprotein a; WBC, white blood cell count ; HGB, hemoglobin. All p values were two-tailed and p < 0.05 was been considered as significant. *p < 0.05
